# Near infra-red fluorescence identification of the thoracic duct to prevent chyle leaks during oesophagectomy

**DOI:** 10.1007/s00464-021-08912-1

**Published:** 2021-12-14

**Authors:** Thomas G. Barnes, Thomas MacGregor, Bruno Sgromo, Nicholas D. Maynard, Richard S. Gillies

**Affiliations:** 1 Nuffield Department of Surgical Sciences, University of Oxford, Oxford, UK; 2 Department of Oesophagogastric Surgery, Oxford University Hospitals NHS Foundation Trust, Oxford, UK

**Keywords:** Fluorescence, Oesophagectomy, Minimally invasive surgery, Thoracic duct leaks, Indocyanine green

## Abstract

**Background:**

Chyle leaks following oesophagectomy are a frustrating complication of surgery with considerable morbidity. The use of near infra-red (NIR) fluorescence in surgery is an emerging technology and the use of fluorescence to identify the thoracic duct has been demonstrated in animal work and early human case reports. This study evaluated the use mesenteric and enteral administration of indocyanine green (ICG) in humans to identify the thoracic duct during oesophagectomy.

**Methods:**

Patients undergoing oesophagectomy were recruited to the study. Administration of ICG via an enteral route or mesenteric injection was evaluated. Fluorescence was assessed using a NIR fluorescence enabled laparoscope system with a visual scoring system and signal to background ratios. Visualisation of the thoracic duct under white light and NIR fluorescence was compared as well as any identification of active chyle leak. Patients were followed up post-operatively for adverse events and chyle leak.

**Results:**

20 patients received ICG and were included in the study. The enteral route failed to fluoresce the thoracic duct. Mesenteric injection (17 patients) identified the thoracic duct under fluorescence prior to white light in 70% of patients with a mean signal to background ratio of 5.35. In 6 participants, a possible active chyle leak was identified under fluorescence with 4 showing active chyle leak from what was identified as the thoracic duct.

**Conclusion:**

This study demonstrates that ICG administration via mesenteric injection can highlight the thoracic duct during oesophagectomy and may be a potential technology to reduce chyle leak following surgery.

**Clinical trial registration:**

Clinical trials.gov (NCT03292757).

Chyle leak is a frustrating complication following oesophagectomy. The reported incidence of chylothorax ranges from 1% to as high as 9% [1–3]. Chyle leak is associated with considerable morbidity namely from chest complications including pneumonia and respiratory failure. High volumes of fluid can be lost from a thoracic duct injury resulting in long durations of indwelling chest drains, hypovolaemia and malnutrition from the loss of fat-soluble vitamins, proteins and electrolytes. There is also a risk of sepsis secondary to lymphopaenia and death following thoracic duct injury [3] with mortality reported as high as 75% [4]. Management is usually non-operative with pleural drainage and dietary change in the form of fat free enteral feed or total parenteral nutrition to allow resolution of the chylothorax which can last several weeks; when unsuccessful, or when large volume leakage occurs, operative intervention is required to localise the leak and, usually, ligate the thoracic duct. Such reoperation in the post-operative setting can be challenging, partly due to variation in thoracic duct anatomy and its accessory channels. Traditional techniques employed to visualise the thoracic duct intraoperatively to facilitate resection or protect from injury include the enteral administration of cream [4] and methylene blue [5]. A difficulty with these techniques is that light reflected from the cream will not penetrate the covering fat and fascia and therefore would only be visible once the thoracic duct is dissected free from surrounding structures. NIR (near infra-red) fluorescence imaging of the duct could allow better visualisation.

To date, thoracic duct fluorescence has been demonstrated by Ashitate et al. [6] who demonstrated in rats and pigs that NIR visualisation is possible with IRDye 800CW-CA and IRDye 800CW conjugated to albumin following subcutaneous groin, thigh or shin injection. They also developed a porcine model of thoracic duct injury where it was possible to visualise a leaking duct as well as clearly outlining accessory channels. In humans, there have been 4 unconvincing case reports of indocyanine green (ICG) use to attempt to identify a chyle leak at re-look thoracotomy [7–10]. The only in vivo report of human use of fluorescence either to highlight the duct or identify chyle leakage has been described by Vecchiato and colleagues where ICG was administered into the lymph nodes in the groin under ultrasound guidance [11].

This study aimed to outline the technique of using ICG for real time near infra-red identification of the thoracic duct.

## Materials and Methods

This study was an IDEAL [12] 1/2a open label prospective proof of principle study assessing ICG to fluoresce the thoracic duct during oesophagectomy conducted in a single tertiary referral centre. Two methods were compared: infiltration of ICG mixed with double cream administered enterally via a feeding jejunostomy and subperitoneal injection of ICG into the small bowel mesentery during the abdominal phase of surgery. ICG was given in the operating room following induction of anaesthesia and fluorescence was assessed using a commercially available fluorescence enabled laparoscope compatible with ICG (PINPOINT or Stryker). The study was approved by Wales Research Ethics Committee 7 (17/WA/0295), sponsored by Oxford University Hospitals NHS Foundation trust and confirmed by the MHRA that clinical trial approval was not required. The study was registered on clinicaltrials.gov (NCT03292757). Fluorescence enabled laparoscopic equipment was provided on loan by Stryker.

All participants over 18 years of age undergoing elective oesophagectomy were eligible to enter the study and provided informed consent after being counselled regarding participation. Participants were excluded if they had a known allergy to iodine or ICG, lactose intolerance, significant liver failure or were pregnant, planning pregnancy or breastfeeding. The study was designed to perform ICG fluorescence initially on 3 participants with each method after which the most successful technique would be further investigated.

Baseline assessments were performed on the day of surgery including body mass index (BMI), medical comorbidity and past surgical history. Following induction of anaesthesia and entering the abdominal cavity, ICG was administered in the following methods:

ICG Cream: 50mL of double cream mixed with 2.5mL of reconstituted ICG (10ml in 25mg of ICG) administered via the feeding jejunostomy either formed pre-operatively as part of neoadjuvant treatment or formed at the end of the abdominal part of the oesophagectomy.

Small bowel mesentery: 1.5–2mL of reconstituted ICG (as above) injected into the small bowel mesenteric root as a bleb just under the peritoneum of the mesentery. A 25 gauge needle was used and the tip advanced just beneath the peritoneum and the ICG instilled. In contrast to the aforementioned groin injection targeting lymph nodes, subperitoneal injection did not target the lymphatics.

Following administration, the operation proceeded as per the standard of care. During the thoracic phase of the procedure, attempts were made at numerous unfixed timepoints to visualise the thoracic duct under white light and then fluorescence. The time point (following administration) was recorded and whether the thoracic duct was visible with either white light alone, fluorescence alone, both modalities or not visible. Finally, following removal of the oesophageal specimen, observation for any chyle leakage was undertaken with white light and fluorescence. This involved assessing for any free chylous appearing liquid in the operating field under white light or fluorescent appearing fluid under NIR. Following these assessments, the participant returned to standard care. At 30 days following surgery the participant’s clinical notes were reviewed for any adverse events.

Signal to background ratio for each visualisation was assessed using Fiji (version 2.0.0-rc-68/1.52 h). Briefly, the image captured at the time of fluorescence assessment was loaded onto the Fiji software, a ‘target’ shape drawn around the area of the thoracic duct and the signal calculated using the ‘measure’ function. The same shape was moved to a background area and the background signal measured in three different places to provide a mean background. Comparison of mean signal and background data was analysed using the student t-test and objective measurements were assessed over time using non-linear logistic regression and analysed using SPSS statistics (IBM, version 22.0). A *P* value of less than 0.05 was deemed statistically significant.

## Results

**Fig. 1:**
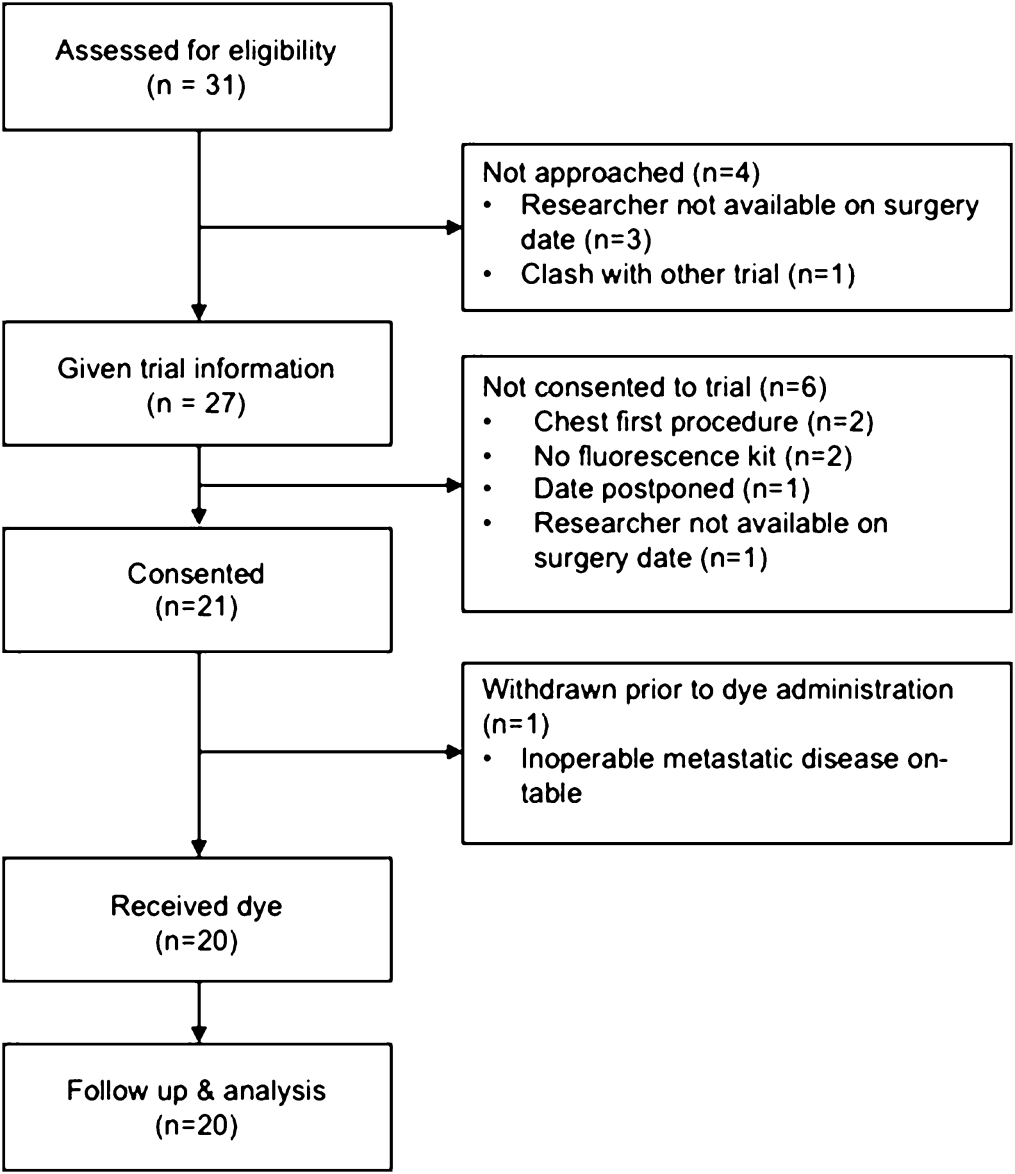
Study flow diagram

Thirty-one subjects were assessed for eligibility and 20 participants received the dye and completed the study (Fig. 1).

The majority of patients were male (*n* = 16) with a median age of 62 years (range of 37–78) with a median BMI of 25.7 (range of 16.95–35.33). All participants underwent planned curative oesophagectomy for cancer by one of three different surgeons with either an open procedure (*n* = 11), a hybrid procedure with laparoscopic abdomen and open thorax (*n* = 6) or minimally invasive oesophagectomy (*n* = 3). Of the open procedures, 7 were left thoracoabdominal resections and 4 were Ivor-lewis oesophagectomies.

### Fluorescence results—feeding jejunostomy

**Fig. 2:**
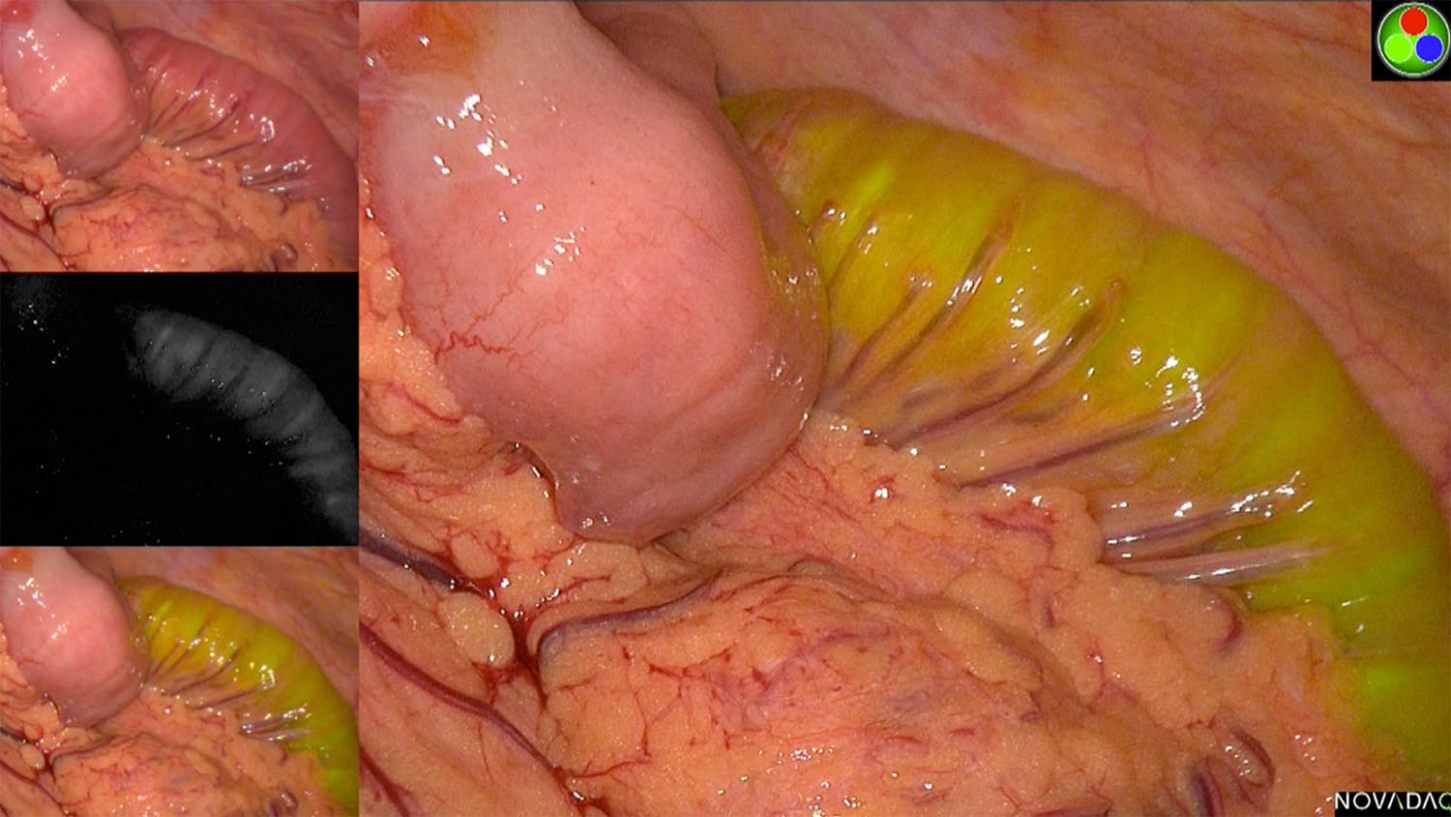
Representative image of fluorescent small bowel following ICG-cream mix infiltrated via a feeding jejunostomy

Three participants received ICG-cream via the feeding jejunostomy. No fluorescent signal was seen at any point in the region of the thoracic duct or in any location throughout the chest during the procedure, despite visualisation of the thoracic duct under white light. The ICG-cream mix fluoresced clearly in the small bowel but was not absorbed into the lymphatics up to 6 h and 59 min following administration (Fig. 2). Administration took between 10 and 16 min to inject the 100 mL of ICG-cream into the feeding jejunostomy. Following a review by the trial steering committee, it was decided that the feeding jejunostomy technique would be abandoned in favour of assessing the more successful mesenteric injection technique in the approved number of patients.

### Fluorescence results—mesenteric injection

**Fig. 3:**
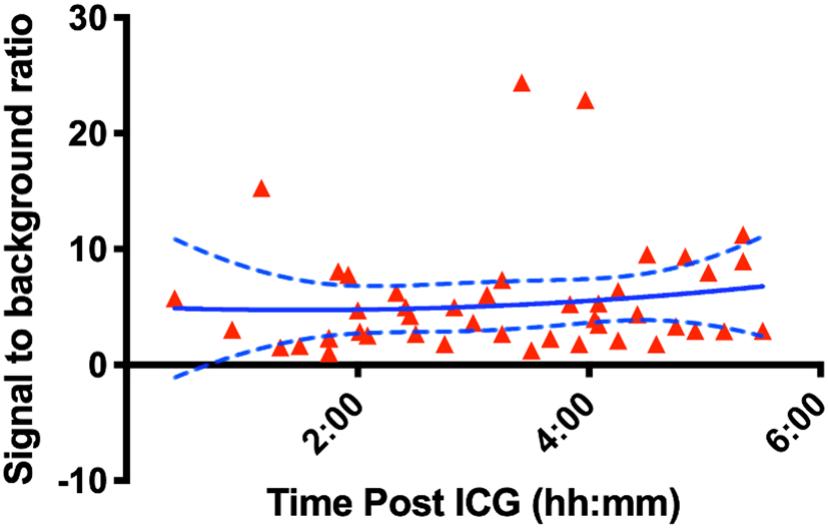
Signal to background ratio over time. Red triangles = individual measurements, solid blue line = mean, dashed blue line = 95% confidence interval (Color figure online)

**Figs. 4:**
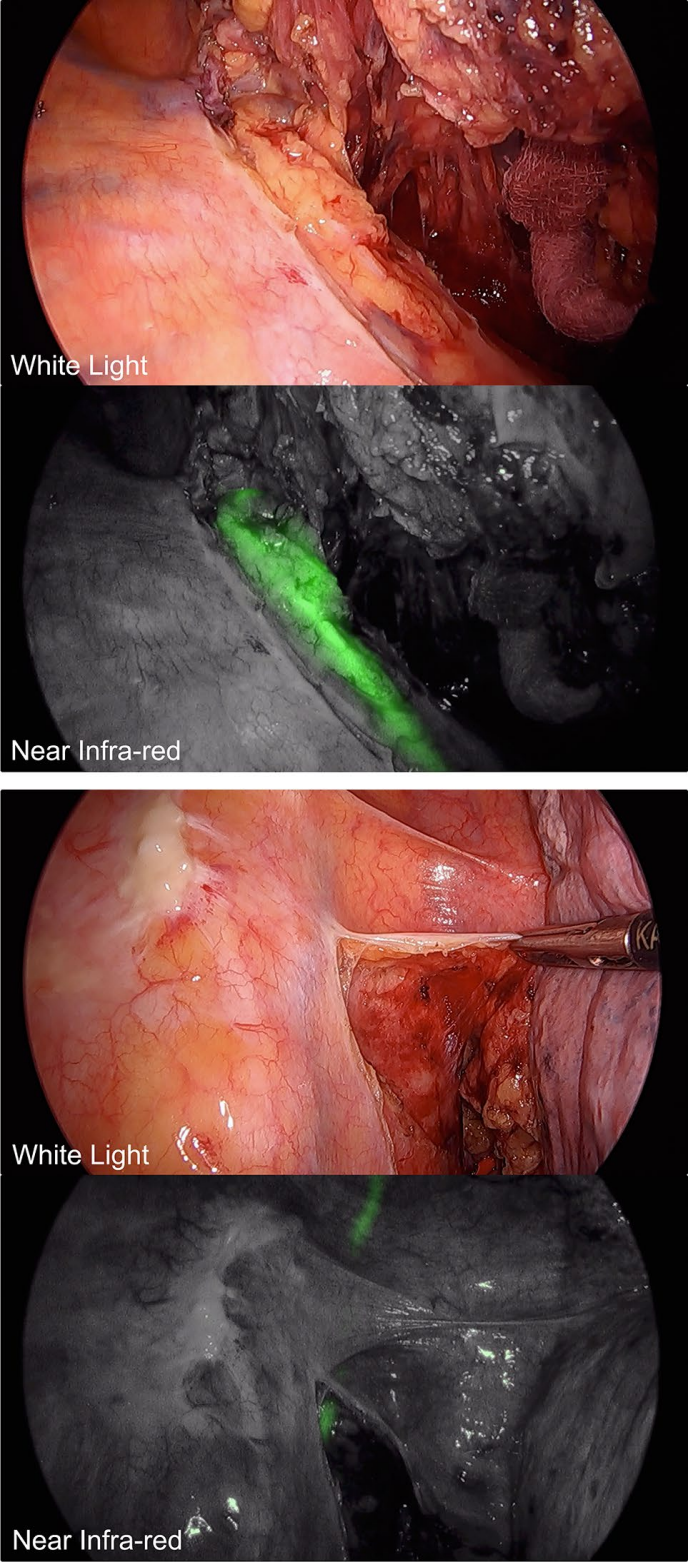
Representative images of white light and near infra-red fluorescence of the thoracic duct during minimally invasive thoracic dissection of the oesophagus. Note that in both images, the thoracic duct cannot be clearly seen under white light but can clearly be seen with fluorescence (green) (Color figure online)

In 17 participants, ninety observations for fluorescence in the thoracic duct were performed from 4 min to 5 h and 30 min (median 2 h 31 min) following ICG administration (Fig. 3). The thoracic duct was visualised with both white light and fluorescence in 12 participants with fluorescence apparent before white light (median = 84 min, range of 10–185 min) and all had a higher subjective score with fluorescence than white light. In 2 participants, the thoracic duct was only seen with fluorescence but not white light and vice-versa in a further 2 participants. The thoracic duct was not visible with either modality in 1 participant. Representative images are seen in Fig.4.

Mean thoracic duct signal was 75.12 (95% CI 62.66–87.58) and mean background signal was 24.18 (95% CI 17.32–31.03) with the signal being significantly higher than the background (*P* < 0.0001). Mean signal to background ratio was 5.354 (95% CI 3.91–6.80) ranging from 1.064 to 24.37. Over the time assessed in this study, the rate of decline was zero (slope 0.4225, 95% CI − 0.678 to 1.523, *P* = 0.443).

### Identification of intraoperative chyle leak

**Fig. 5:**
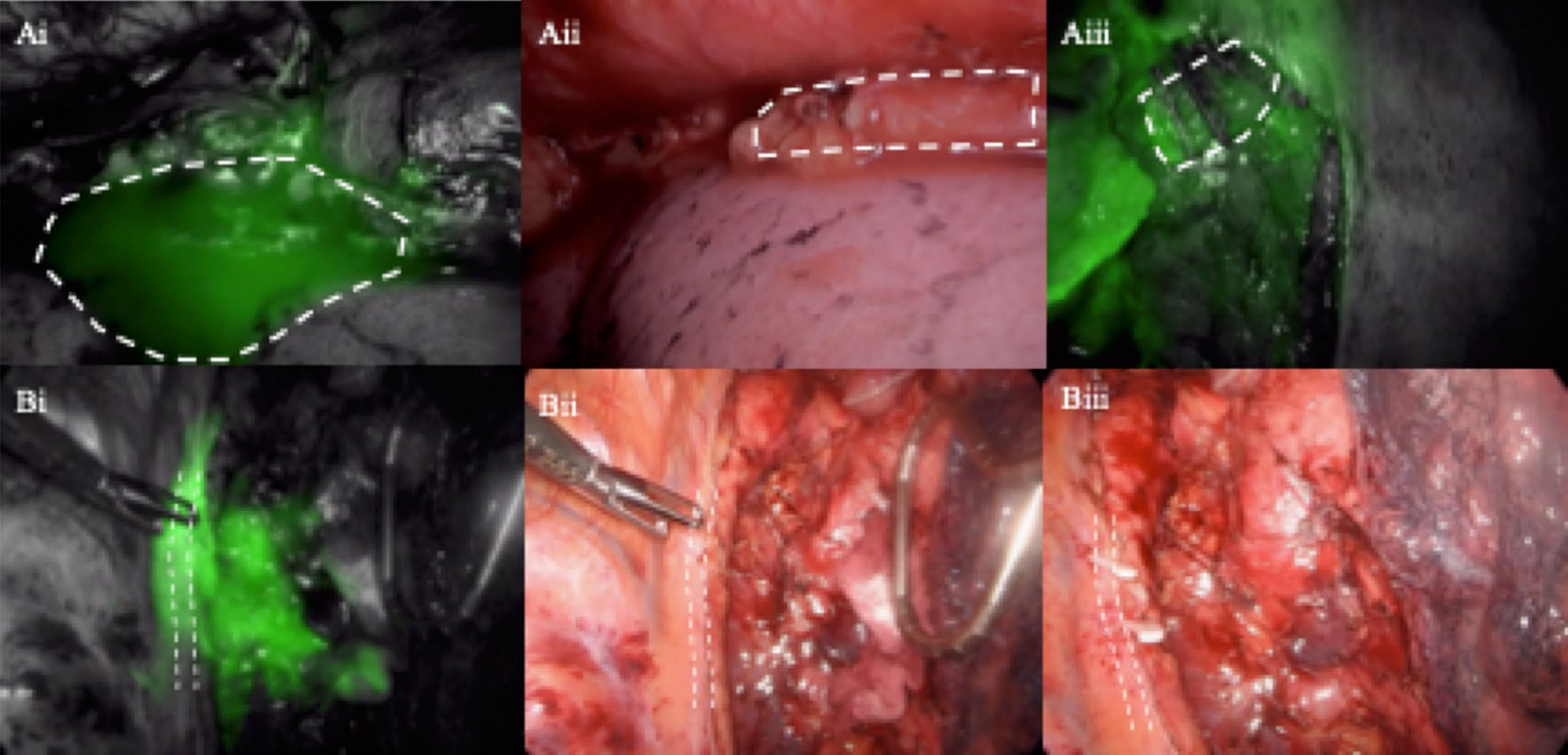
Representative images of identifying chyle leak under fluorescence in two patients. **A** and **B** represent two separate participants. **Ai**—image under fluorescence identifying fluorescent ‘pool’ of fluid (white line). **Aii**—image with white light outlining area of thoracic duct. **Aiii** clipped thoracic duct under fluorescence. **Bi** = fluorescent image outlining (white line) thoracic duct and adjacent to this fluorescent fluid. **Bii**—white light image of **Bi**. **Biii—**white light image following clipping of thoracic duct

In 6 of the 17 participants receiving ICG via mesenteric injection, fluorescent fluid was identified within the operative field following specimen removal. These were all identified by fluorescence only. Of these six, four had fluorescent fluid emerging from a fluorescent tubal structure identified as the thoracic duct. In all four of these participants the main thoracic duct was further clipped, wash applied to the thorax and re-imaging of fluorescence revealed no further leakage. In the other two participants, fluorescent fluid was seen more diffusely throughout the operative field with one resulting in no further action being taken and in the other a small leaking branch was identified and clipped. Representative images are shown in Fig. 5. Five of these participants were discharged without any complication within 10 days. One patient suffered an anastomotic leak and remained in hospital for 40 days post-operatively.

#### Follow-up and postoperative chyle leak

All patients were followed up for 30 days following ICG administration. No participants suffered adverse reactions related to ICG. Median length of stay was 10 days (range of 7–40 days) with one participant having a possible low volume chyle leak identified with chylous appearing liquid in the drain; however, their drain was removed after 5 days (2 days longer than standard drain removal in our unit) with no further complications and the patient was discharged on day 8 post-operatively.

## Discussion

This proof of principle study is the first to successfully report the use of fluorescence in thoracic duct visualisation and identification of chyle leak intraoperatively during oesophagectomy. Mesenteric injection of ICG is a simple, safe and effective technique and it may identify and potentially prevent chyle leaks. Two techniques were evaluated: enteric administration using ICG-cream mixture and small bowel mesentery injection. Enteric administration was not successful in thoracic duct identification. In contrast, using mesenteric injection the thoracic duct was visualised in 15 of 17 subjects with 2 of these being only visible under fluorescence; failure of the technique was observed in only 2 subjects. Fluorescent signal was seen over 5 h after administration indicating that repeat administration is unlikely to be required. The S2B (signal to background) ratio was always over 1 and an average of 5. This high signal to background ratio indicates that the thoracic duct would be clearly visible and distinguishable from the minimal background signal observed in the study.

Prior to exploring enteric administration in vivo, in preliminary, non-published work, it was demonstrated that fluorescence of ICG when mixed with acidic solution did not exhibit fluorescence properties; however, when mixed with cream prior to addition of acidic solution, fluorescence was demonstrated. It was believed that acidic solutions denatured the molecule such to inhibit fluorescence. ICG is known to bind to proteins and thus, when mixed cream, retained its fluorescence properties. It was therefore believed that as fat is rapidly absorbed from the bowel, it would eventually reach the thoracic duct and exhibit the desired fluorescence. Longer time may be required for this absorption to take place; however, assessment of this was outside the protocol of this study.

Practice amongst surgeons varies with regard to the management of the thoracic duct during oesophagectomy and depends on the extent of lymphadenectomy [13] and whether to routinely resect the thoracic duct. Advocates of routine resection of the thoracic duct point to the demonstrated presence of lymph nodes in the fatty tissue surrounding the thoracic duct [14, 15] although Udagawa and colleagues found no significant difference in survival when it was resected. A study comparing outcomes after resection of the thoracic duct and preservation of the thoracic duct showed a significantly higher yield of lymph nodes and a possible improvement in recurrence free survival when the thoracic duct was resected, with the caveat that more patients in the thoracic duct resection arm received chemotherapy [16]. Opponents of routine thoracic duct resection point to possible complications including chylothorax, increased operating time and blood loss. The technique that we have described to allow fluorescence of the thoracic duct would be potentially useful to all surgeons whether they routinely resect the thoracic duct or leave it in situ*,* not only to identify the duct to aid resection but also to identify chyle leakage which can occur with either approach.

There are two published reports of ICG fluorescence in the thoracic duct at the time of primary operation. Steffey et al. used a technique of ICG injection into either a popliteal LN (lymph node), mesenteric LN or mesenteric root in 15 dogs and achieved optimal visibility with mesenteric LN or root injection [17]. They also identified a tear in the thoracic duct which was only highlighted by NIR imaging. In humans, Yang and colleagues utilised ICG fluorescence in 4 patients who underwent re-operative surgery for chyle leak and in all patients the location of the leak was identified with fluorescence prior to white light [18]. ICG was injected bilaterally into the inguinal region as access to the abdomen was not available. We did not evaluate this method, which is a limitation of our technique as the abdomen would not be easily accessible in reoperative thoracotomy. Three further case reports also demonstrate the potential use of ICG to help identify a chyle leak at the time of re-operative surgery [7, 8, 10].

The only other study to successfully utilise ICG as a NIR marker of the thoracic duct [11] utilised the inguinal lymph nodes as the injection site. Whilst the results of their study were promising (19 out of 20 patients with positive NIR thoracic duct visualisation), the authors do not compare NIR visualisation with white light or demonstrate its use in demonstrating active chyle leak at the end of the operation as we do in this study. In addition, the lymph node injection could be quite cumbersome to perform requiring ultrasound guidance with the potential for damaging lymph nodes or inadvertent vascular injury or injection. Subperitoneal injection is technically simpler as it does not require direct targeting of lymphatics.

These studies together with our findings clearly demonstrate that there is a potential clinical use for this technology during transthoracic oesophagectomy. Further work should be undertaken to establish the reproducibility of this technique in other centres before developing guidance on its possible implementation in routine clinical practice.
